# The distinct role of IL-34 and IL-35 in gastric cancer

**DOI:** 10.3389/fimmu.2025.1559508

**Published:** 2025-05-09

**Authors:** Zhiyun He, Jie Dang, Xiang Cui, Bo Li, Shisan Bao, Jingchun Fan

**Affiliations:** ^1^ General Surgery Department, The First Hospital of Lanzhou University, Lanzhou, Gansu, China; ^2^ Central Sterile Supply Department, Second Hospital of Lanzhou University, Lanzhou, Gansu, China; ^3^ Scientific Research Division, The Third Affiliated Hospital of Gansu University of Chinese Medicine, Baiyin, China; ^4^ Scientific Research Division, The First People’s Hospital of Baiyin, Baiyin, Gansu, China; ^5^ School of Public Health, Centre for Evidence-based Medicine, Gansu University of Chinese Medicine, Lanzhou, Gansu, China

**Keywords:** IL-34, IL-35, gastric cancer, TAMs, prognosis

## Abstract

Gastric cancer (GC) remains a major challenge due to its high mortality and morbidity, despite extensive research. Dysregulated host immunity plays a critical role in carcinogenesis, particularly among susceptible cohorts. In the gastric mucosa of GC patients, a reduction in IL-34 and TAM1, accompanied by an increase in TAM2 *via* M-CSF, enhances Th2 cell function, reduces pro-inflammatory activity, and elevates anti-inflammatory responses. Consequently, TAM2 acts in both paracrine and autocrine manners to polarize and boost TAM2, creating a tumour-favourable microenvironment that supports GC progression. High levels of TAM2, observed during advanced GC stages, suppress gastric IL-34 production, further promoting GC development. In contrast, IL-35, a cytokine involved in immune regulation and suppression, is produced by activated T cells and/or B cells in the affected gastric mucosa. Persistent *H. pylori* infection in GC tissues is associated with significant infiltration of IL-35-producing B cells and regulatory T cells (Tregs), which enhance the immunosuppressive and pro-tumour microenvironment by disrupting the local immune balance. Upregulated mucosal IL-35 promotes the polarization of TAM2 and Tregs while suppressing TAM1 cells, fostering a tumour-friendly environment that allows transformed gastric mucosal cells to evade immune surveillance, particularly in chronic *H. pylori*-infected patients. This cascade enhances proliferation and invasion while suppressing differentiation and apoptosis of GC cells. Together, the differential regulation of these cytokines creates an environment that supports cancer progression and resistance to therapy. Targeting the IL-34 and IL-35 pathways may offer a novel therapeutic strategy for improving outcomes in GC patients.

## Gastric cancer

Gastric cancer (GC) remains a significant clinical challenge as the fifth most common cancer worldwide (1). The incidence of GC is highest in Eastern Asia—particularly in Japan, Mongolia, Korea, and China—where it ranks second in cancer prevalence (2). While the age-standardized incidence of GC has gradually declined due to widespread use of non-invasive screening methods, such as the rapid urease test for *Helicobacter pylori* (*H. pylori*) detection (3), the global burden remains substantial. Recent estimates report approximately 1.1 million new cases and 770,000 deaths annually, with incidence rates in Asia nearly twice as high in males as in females (2). Alarmingly, projections suggest that by 2040 the global burden of GC could rise to 1.8 million new cases and 1.3 million deaths annually (2).

The mechanisms underlying GC progression and metastasis, particularly in genetically susceptible populations, remain poorly understood. Such reports highlight the urgent need for improved therapeutic strategies, particularly those targeting immune pathways. Despite advancements in treatment, GC remains the third leading cause of cancer-related deaths globally, with late-stage diagnoses and limited options for advanced disease being primary contributors to its poor prognosis (4). Early detection significantly improves outcomes, yet many patients are diagnosed at advanced stages, resulting in low five-year survival rates. However, it is challenging due to subtle and nonspecific symptoms, which often leads to late-stage diagnoses with poor prognosis. Even in early-stage cases, metastasis dramatically worsens prognosis (5).

The stunning discovery by Drs Marshall and Warren in 1984 identified *H. pylori* infection as a key factor in the development of (GC), revolutionizing its management with antibiotics and acid-reducing medications (6). *H. pylori* induce chronic gastric mucosal inflammation, also known as chronic gastritis. Although *H. pylori* affect half the global population, only some develop chronic gastritis or cancer (7). Host mucosal immunity plays a pivotal role in GC progression, involving both cellular and humoral immune responses, as well as a delicate balance of pro- and anti-inflammatory cytokines in the gastric mucosa (12). The host’s gut-associated lymphoid tissue (GALT) and immune responses help eliminate pathogens but compromised immunity in some individuals can still lead to persistent infection, which contributes to mucosal cell transformation and GC (9–11). *H. pylori* infection and high-salt diets are major contributors to GC, particularly in Asia, and the lower GC incidence in females has been attributed to the protective effects of their hormones (7, 8).

Whilst the gastric mucosa possesses intrinsic regenerative capabilities, persistent stimulation under chronic conditions, such as chronic gastritis, can lead to the transformation of damaged epithelial cells. Ordinarily, transformed cells are eliminated by host immune surveillance mechanisms (13). However, they can evade detection by secreting immunosuppressive factors, thereby facilitating tumour progression, especially in genetically predisposed individuals (14). Host immunosurveillance, including cellular and humoral immunity, plays a critical role in cancer suppression. Pro-inflammatory cytokines (IL-34) and anti-inflammatory cytokines (IL-35) along with tumour-associated macrophages affects the development of GC. Thus, a deeper understanding of the interplay between host immunity and tumorigenesis is essential for developing more effective treatment strategies for GC. To illustrate the relationship among these factors, the relevance connection is going to be presented below.

## IL-34 – pro-inflammatory cytokine

IL-34, a cytokine discovered in 2008 through computational and experimental approaches, shares its receptor with macrophage colony-stimulating factor (M-CSF) (15). Encoded by a gene on chromosome 16, IL-34 signals through the colony-stimulating factor-1 receptor (CSF-1R), a pathway it shares with M-CSF (15). Despite structural differences, the two cytokines exhibit functional overlap, advancing our understanding of cytokine networks and receptor-ligand interactions in the immune system (16).

Structurally, IL-34 and M-CSF share similarities in their active sites and demonstrate tissue-specific functions under pathological conditions (17, 18). IL-34 is prominently expressed in the brain, skin, and spleen, where it regulates the development and function of myeloid cells, including macrophages and dendritic cells (15). The CSF-1/CSF-1R pathway is critical for hematopoietic cell differentiation, growth, maintenance, and survival, as shown in both *in vitro* and *in vivo* studies (19, 20). Immunologically, IL-34 promotes the survival, differentiation, and proliferation of monocytes, macrophages, and other myeloid cells (17). It plays a pivotal role in innate and adaptive immune responses, tissue repair, and homeostasis. IL-34 also supports the development of Langerhans cells and enhances the activation of effector T cells, particularly Th17 cells (16, 21). Furthermore, it recruits macrophages and stimulates the production of pro-inflammatory cytokines.

At the pathophysiological level, IL-34 is implicated in various diseases, including autoimmune and inflammatory disorders. Elevated IL-34 expression has been observed in inflammatory bowel disease in both human and animal models (22). In neuroinflammatory conditions like Parkinson’s disease, IL-34 modulates microglial cells, contributing to localized inflammation through autocrine and paracrine mechanisms (17).

IL-34 plays dual roles in cancer development. On one hand, it promotes tumour progression by recruiting tumour-associated macrophages (TAMs) that facilitate immune evasion and tumour growth, partly through mechanisms such as p35 inactivation (23). On the other hand, IL-34 enhances anti-tumour immunity by activating effector T cells and promoting macrophage-mediated tumour cell killing (24). Understanding IL-34’s complex roles in both physiological and pathological processes is crucial for developing targeted therapies and improving patient outcomes. Continued research into its diverse functions could provide critical insights for therapeutic interventions in diseases such as cancer, autoimmune disorders, and neurodegenerative conditions.

## IL-35 – anti-inflammatory cytokine

IL-35 was discovered in 2007 during investigations into the immunosuppressive mechanisms of regulatory T cells (Tregs) (25). It is a heterodimeric cytokine composed of two subunits: p35, shared with IL-12, and Epstein-Barr virus-induced gene 3 (EBI3), shared with IL-27 (25). This shared subunit structure classifies IL-35 as part of the IL-12 cytokine family.

Primarily produced by Tregs (26), IL-35 functions as an anti-inflammatory cytokine, suppressing effector T cells (Teffs) and promoting Treg expansion to maintain immune tolerance (27). Previously, Treg-mediated immunosuppression was attributed mainly to contact-dependent mechanisms and cytokines such as IL-10 and TGF-β. However, the discovery of IL-35 added complexity to these pathways, establishing it as a crucial mediator of immune suppression and a key factor in preventing autoimmune diseases. By inhibiting Teff proliferation, IL-35 prevents excessive inflammatory responses that could lead to tissue damage or autoimmunity (28). Thus, IL-35 plays a vital role in maintaining immune homeostasis.

It has been reported that, in autoimmune and inflammatory diseases, enhancing IL-35 activity has shown therapeutic potential by mitigating excessive immune responses and promoting immune tolerance. For example, IL-35 inhibits Teff proliferation, alleviating overactive immune responses in psoriasis (28). Similarly, it suppresses autoreactive immune cells and supports regulatory pathways in systemic lupus erythematosus (29). IL-35 has also been shown to reduce disease severity in rheumatoid arthritis, further highlighting its potential as a therapeutic target (30). IL-35 also protects host tissues by limiting excessive inflammation in conditions such as inflammatory bowel disease (25). Additionally, it plays a protective role in vascular inflammation, potentially preventing the progression of atherosclerosis by suppressing inflammatory responses (31). However, its role in infectious diseases is more complex. Elevated IL-35 levels can suppress antiviral responses, facilitating viral persistence in chronic hepatitis B and C infections (32).

IL-35 shifts from being protective to promoting immune evasion in cancers. IL-35 creates a tumour-friendly microenvironment by suppressing anti-tumour immune responses, enabling cancer cells to proliferate and metastasize (33). Within the tumour microenvironment (TME), IL-35 and IL-10 work synergistically to drive T cell exhaustion by regulating inhibitory receptor expression and shaping exhaustion-associated transcriptomic profiles of CD8^+^ tumour-infiltrating lymphocytes (TILs) (34). While IL-10 predominantly affects effector T cell fate, IL-35 influences memory T cell differentiation. Together, these cytokines suppress anti-tumour immunity, facilitating tumour growth and immune evasion (34).

Thus, understanding the multifaceted roles of IL-35 is critical for developing targeted interventions. Future research should aim to elucidate the context-dependent effects of IL-35, particularly its dual roles in immune suppression and activation, to harness its therapeutic potential in both autoimmune diseases and cancer.

## Tumour-associated macrophages

As noted above, host immunity both promotes and inhibits the development of cancer. Tumour-associated macrophages (TAMs) may contribute to the host’s defence against transformed cells and can also support tumour growth. It was initially reported that macrophages infiltrate and/or surround tumours in significant numbers, potentially assisting lymphocytes in killing cancer cells. These macrophages are express the pan-macrophage marker CD68 (35). However, the functions of TAMs remain controversial, particularly regarding whether they are pro-tumorigenic or anti-tumorigenic in cancer pathogenesis (36). TAMs are often classified into two subtypes, TAM1 and TAM2, based on their polarization *in vitro* and the expression of specific surface biomarkers (37).

Macrophage activation is dynamic rather than static. During inflammatory conditions or tissue damage, M1 macrophages can transition into M2 or M2-like cells as inflammation progresses or resolves (36). Functionally, TAM1 macrophages, driven by Th1 responses, are associated with macrophage-mediated tissue damage and tumour cell destruction, playing key roles in initiating and sustaining inflammation. These macrophages are pro-inflammatory and exhibit anti-malignant activity through the production of various inflammatory mediators. Conversely, TAM2 macrophages, driven by Th2 responses, contribute to tissue repair and remodelling. These macrophages are anti-inflammatory, suppressing host immunity and thereby promoting malignant cell proliferation and progression (36).

More recently, studies have reported that TAMs express PD-1, which inhibits phagocytosis and suppresses tumour immunity (38). PD-L1 expression by TAMs has also been observed, based on cross-tissue single-cell analyses of human monocytes and macrophages in both healthy and diseased states (39).

However, recent research challenges the strict classification of TAMs into TAM1 or TAM2 based solely on surface biomarkers as their functional roles in cancer are highly context-dependent. For instance, TAMs often exhibit overlapping behaviours, with TAM2 macrophages increasing while TAM1 macrophages are suppressed, collectively promoting tumour progression (40, 41). This complexity emphasises the need for a deeper understanding of the functional plasticity of TAMs in the tumour microenvironment.

We have highlighted the differential roles of IL-34 and IL-35 and their relationship with TAMs during the development of GC and emphasized their potential as therapeutic targets. We have considered the association between key factors, including anti-inflammation and pro-inflammatory cytokines, and the polarisation of macrophages, influential in the development of GC *via* GM-CSF and/or PD-1/PD-L1 pathways. Future research should focus on exploring the mechanisms through which these cytokines contribute to GC progression and delving into strategies that harness their therapeutic potential for clinical benefit.

## IL-34 in GC

### IL-34 expression in gastric mucosa and GC progression

First from clinic immunological point of view, it has been reported that IL-34, expressed on the mucosal epithelial cells of non-cancerous gastric tissues, is significantly suppressed in the gastric mucosa of GC patients (42). Furthermore, IL-34 expression in gastric mucosal epithelial cells is inversely correlated with GC differentiation (42), suggesting an important suppressive role of IL-34 in GC development. However, this protective effect may be diminished in susceptible individuals facing intrinsic or extrinsic challenges, ultimately leading to GC. Higher IL-34 expression in the gastric mucosa is associated with better prognosis in female GC patients compared to males (42), possibly due to hormonal protection. Conversely, older GC patients exhibit higher IL-34 levels than younger patients, potentially reflecting reduced vulnerability to malignancy in older cohorts (43). However, no significant differences in IL-34 expression were observed between patients with varying tumour sizes or metastasis statuses, possibly due to study limitations such as small sample sizes or single-centre designs.

Interestingly, while IL-34 appears to have a suppressive role in GC, it promotes TAM2 differentiation and exhibits a pro-tumoral role in colorectal cancer (44). This functional divergence may arise from differences in microbial loads and gut-associated lymphoid organ regulation between the upper and lower gastrointestinal (GI) tract. Similar differential roles have been reported for cytokines such as IL-37 and IL-38 (45, 46).

### Mechanistic insights into IL-34 and TAMs

From mechanistic point of view, it has been reported that IL-34 supports macrophage maturation and function through the CSF-1 receptor on monocytes (47). Paradoxically, GC tissues exhibit increased infiltration of TAMs, raising the question of the role of IL-34 in this process. This discrepancy could be attributed to IL-34’s efficacy in recruiting monocytes and driving their differentiation into TAMs, particularly favouring the TAM2 subset.

Although the specific subset of TAMs in GC mucosa remains unconfirmed (42), a positive correlation between TAMs infiltration and GC differentiation and invasion suggests that TAM2 may predominate (48). TAM2 exhibits pro-tumoral characteristics, promoting GC cell proliferation and invasion while inhibiting differentiation and apoptosis (42). Additionally, TAM2 may enhance GC progression by secreting VEGF and other factors that stimulate proliferation and invasion (49). Recent evidence indicates that IL-34 facilitates tumour immune evasion by coordinating TAMs to inactivate p53 (23). This highlights IL-34’s dual role in tumorigenesis, warranting further investigation.

### Clinical and research implications

From clinical application point of view, IL-34 expression is inversely correlated with GC differentiation and TNM stage, aligning with a 5-year survival rate. However, IL-34 is not an independent prognostic factor (42). Future multi-centre studies with larger, diverse cohorts are necessary to validate these findings. A significant correlation between IL-34/CD68+ TAMs and GC prognosis suggests their synergistic protective role. High IL-34 and CD68+ TAM levels are associated with the best prognosis, while low levels of both markers indicate the worst outcomes (42). These findings highlight the potential of IL-34/CD68^+^ TAMs as prognostic biomarkers and therapeutic targets. Reduced gastric IL-34 exacerbates GC progression, forming a self-reinforcing loop that fosters a tumour-promoting environment. Understanding IL-34’s complex roles in GC could pave the way for novel therapeutic strategies tailored to patient-specific profiles.

**Figure 1:**
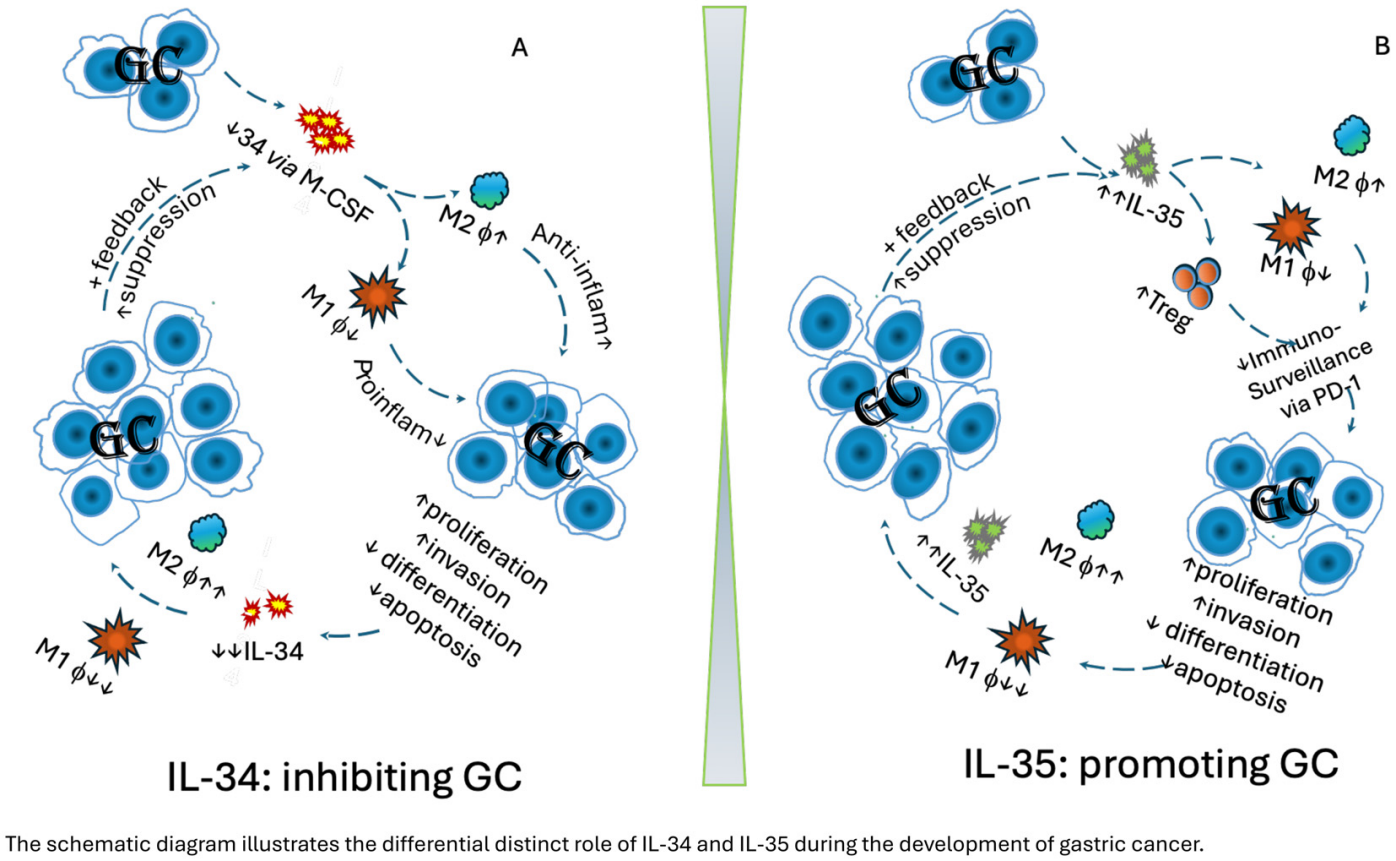
The schematic diagram illustrates the differential distinct role of IL-34 and IL-35 during the development of gastric cancer. **(A)** Protective role of IL-34 in gastric cancer:.Decreased intra-tumoral IL-34 leads to reduce polarisation of in M1 *via* GM-CSF pathway, but promote M2 macrophages. Consequently, anti-inflammatory cytokines are elevated, whereas pro-inflammatory cytokines are reduced, promoting proliferation and invasion of gastric cancer, while supressing differentiation and apoptosis of GC cells. Consequently, this leads to a pro-tumoral environment. The further development of GC continuously suppresses polarisation of M1 in the tumour, and further supresses intra tumoral IL-34 production. This, in turn, enhances GC progression, creating a feedback loop that further drives tumour development. **(B)** IL-35 in gastric cancer. Increased intra-tumoral IL-35 contributes to reduce polarisation of M1, but promote M2 macrophages, as well increase Treg cells. This leads to compromised host immunosurveillance *via* the PD-1/PD-L1 pathway, promoting GC development, e.g. GC proliferation, invasion, while decreasing differentiation and apoptosis. As GC continues to develop, IL-35 elevation intensifies, further reducing M1 macrophages while promoting M2 macrophages, leading to even higher IL-35 levels. This, in turn, accelerates GC progression, creating a feedback loop that further drives tumour development.

The schematic diagram generated further illustrates this mechanism: In the gastric mucosa of GC patients, a reduction in IL-34 and TAM1, accompanied by an increase in TAM2 via M-CSF, enhances Th2 cell function, reduces pro-inflammatory activity, and elevates anti-inflammatory responses. Consequently, TAM2 acts in both paracrine and autocrine manners to polarize/boost TAM2, creating a tumour-favourable microenvironment that supports GC progression. High levels of TAM2, observed during advanced stages of GC, suppress gastric IL-34 production, further promoting GC development (**Figure 1A**).

### Future directions

First, validating IL-34 as a diagnostic and/or prognostic target through large, multicentre cohorts. Second, investigating the efficacy and safety of IL-34 inhibitors in preclinical and clinical trials, as well as examining the potential influence of sex hormones on IL-34 expression and GC progression to better understand its differential impact in males and females. Third, while some GC patients benefit from targeted immunotherapies, leading to prolonged survival, others experience compromised outcomes, highlighting the need for further investigation into this variability. Furthermore, studies should employ single-cell omics and humanized GC models to characterize TAM subsets and their functions in GC. Finally, precision medicine strategies could utilize engineered macrophages to refine therapeutic interventions (50).

## IL-35 in GC

### IL-35’s role in immune regulation and tumour promotion

As previously discussed, IL-35, a cytokine involved in immune regulation and suppression, is produced by activated T cells and/or B cells in the affected gastric mucosa (51). Persistent *H. pylori* infection in GC tissues is associated with significant infiltration of IL-35-producing B cells and regulatory T cells (Tregs), which enhance the immunosuppressive and pro-tumour microenvironment by disrupting the local immune balance. Upregulated mucosal IL-35 promotes the polarization of TAM2 and Tregs while suppressing TAM1 cells, fostering a tumour-friendly environment that allows transformed gastric mucosal cells to evade immune surveillance, particularly in chronic *H. pylori*-infected patients. This cascade enhances proliferation and invasion while suppressing differentiation and apoptosis of GC cells.

The synchronized effects of IL-35 and TAM2 further exacerbate tumour progression by establishing a self-reinforcing loop that suppresses TAM1 cells while elevating IL-35 and TAM2 levels. Studies have shown that elevated IL-35 levels may represent a compensatory response to suppress uncontrolled inflammation caused by *H. pylori* infection. However, this response inadvertently promotes tumorigenesis by suppressing anti-tumour immunity (52). Thus, IL-35 plays a pivotal role in GC by undermining the host’s anti-tumour immune defences.

### Mechanisms of IL-35 in GC progression

From mechanistic point of view, IL-35 drives GC progression through several mechanisms. First, it enhances Treg activity by promoting their expansion and suppressive functions, which inhibit effector T cells (Teffs) and maintain an immunosuppressive microenvironment conducive to tumour growth. Elevated levels of Tregs in GC tissues correlate with tumour progression and poor survival outcomes (53). Second, IL-35 induces exhaustion in CD8^+^ tumour-infiltrating lymphocytes (TILs) by increasing the expression of inhibitory receptors, reducing their cytotoxic capacity and facilitating tumour growth (34). This is supported by evidence showing a positive correlation between IL-35 and Ki67 expression, linking IL-35 to GC cell proliferation—a hallmark of cancer progression (54). Additionally, IL-35 promotes the expression of anti-apoptotic proteins like Bcl-2, enhancing tumour cell survival and resistance to apoptosis (54). Finally, IL-35 contributes to angiogenesis, a critical process in neoplasm invasion and metastasis. It alters the expression of angiogenesis regulators such as TIMP1, PAI1, and IGFBP1 to promote blood vessel formation (54). Tumour-derived IL-35 also facilitates the accumulation of myeloid-derived suppressor cells (MDSCs) and TAM2, which further enhance angiogenesis, cancer progression, and metastasis (53). These findings underscore IL-35’s multifaceted role in driving GC progression and its potential as a therapeutic target.

### IL-35 and immune checkpoints

It is well known that programmed cell death protein 1 (PD-1), an immune checkpoint receptor expressed on activated T cells, regulates immune tolerance. Tumour cells exploit this pathway by overexpressing PD-L1, allowing them to evade immune detection and enhance survival. PD-1 expression on TAMs also impairs their phagocytic function, but this dysfunction can be reversed with anti-PD-1 antibodies, improving cancer patient outcomes (38). Combining IL-35 blockade with checkpoint inhibitors may restore immune function and enhance therapeutic efficacy (34).

Thus, targeting TAMs and MDSCs, which are recruited and activated by IL-35, is another promising strategy. Therapies aimed at these cells, particularly in combination with IL-35 inhibitors, could effectively disrupt the tumour-promoting microenvironment (40). Similar findings in colorectal cancer (CRC) support this approach, as an inverse correlation between IL-38 and PD-1 expression in CRC lymph nodes has been observed (55). IL-38 appears protective against CRC development (56), contrasting with PD-1’s association with pro-tumour activity (38).

### Clinical implications of IL-35

Considering IL-35 expression in GC correlates with advanced TNM stages, tumour size, and invasion depth (57), these adverse outcomes likely reflect IL-35’s ability to suppress both local and systemic immunity against GC. Elevated circulating IL-35 levels inversely correlate with overall and progression-free survival, indicating its potential as an independent prognostic factor (58). Additionally, circulating IL-35 could serve as a non-invasive biomarker for early GC detection and progression monitoring, particularly in *H. pylori*-infected patients. Emerging evidence also suggests that IL-35 contributes to chemotherapy resistance by promoting GC cell survival through enhanced anti-apoptotic mechanisms, thereby reducing chemotherapy susceptibility (54).

To further illustrate this mechanism, a schematic diagram is also generated: Increased intra-tumoral IL-35 suppresses polarisation of M1, but promotes M2 macrophages, as well promotes Treg cells development. Consequently, these actions lead to compromised host surveillance via the PD-1/PD-L1 pathway, promoting GC development, e.g. GC proliferation, invasion, while decreasing differentiation and apoptosis. During further development of GC, IL-35 elevation intensifies, further reducing M1 macrophages while promoting M2 macrophages, leading to even higher IL-35 levels. This, in turn, accelerates GC progression, creating a feedback loop that further drives tumour development (**Figure 1B**).

### Future directions

For potential clinical direction, targeting IL-35 in combination with therapies like PD-1/PD-L1 inhibitors, CAR-T cell therapy, or anti-angiogenic agents holds promise for improving GC treatment outcomes. Additionally, nanotechnology-based delivery systems for IL-35 inhibitors or TAM modulation could offer a precise treatment method (40). Future research should focus on exploring IL-35’s role in Treg activity, angiogenesis, and tumour proliferation, validating it as a diagnostic and prognostic marker through large, multi-centre cohorts, and assessing IL-35 inhibitors’ efficacy in clinical trials. Further investigation is needed into the impact of sex hormones on IL-35 expression and GC progression, as well as the variability in outcomes observed with targeted immunotherapies.

## Conclusion

We conclude that IL-34 and IL-35 play distinct but significant roles in the pathogenesis of gastric cancer by modulating the tumour-friendly microenvironment through immune regulation. Reduced gastric IL-34 promotes tumour growth by recruiting TAM2 cells to the GC mucosa *via* M-CSF, fostering a tumour-permissive environment. In contrast, elevated IL-35 contributes to immune evasion by promoting Treg cell expansion but suppressing effector T cells. Together, the differential regulation of these two cytokines creates an environment that supports cancer progression and resistance to therapy. Targeting the IL-34 and IL-35 pathways could offer a novel therapeutic strategy to improve outcomes in gastric cancer patients. Further research is needed to clarify their precise mechanistic roles in tumour progression and immune regulation, which could potentially pave the way for cytokine-targeted treatments.

## References

[1] Siegel RL Giaquinto AN Jemal A. Cancer statistics, 2024. CA Cancer J Clin. (2024) 74:12–49. doi: 10.3322/caac.21820 38230766

[2] Morgan E Arnold M Camargo MC Gini A Kunzmann AT Matsuda T. The current and future incidence and mortality of gastric cancer in 185 countries, 2020-40: A population-based modelling study. EClinicalMedicine. (2022) 47:101404. doi: 10.1016/j.eclinm.2022.101404 35497064 PMC9046108

[3] Vaira D Vakil N Gatta L Ricci C Perna F Saracino I. Accuracy of a new ultrafast rapid urease test to diagnose Helicobacter pylori infection in 1000 consecutive dyspeptic patients. Aliment Pharmacol Ther. (2010) 31:331–8. doi: 10.1111/j.1365-2036.2009.04196.x 19891666

[4] Smyth EC Nilsson M Grabsch HI van Grieken NC Lordick F. Gastric cancer. Lancet. (2020) 396:635–48. doi: 10.1016/S0140-6736(20)31288-5 32861308

[5] Lee H Song KY Lee HH Lee J. Worse survival of patients with T1 stage II gastric cancer following radical gastrectomy. J Gastric Cancer. (2023) 23:598–608. doi: 10.5230/jgc.2023.23.e40 37932226 PMC10630564

[6] Marshall BJ Warren JR. Unidentified curved bacilli in the stomach of patients with gastritis and peptic ulceration. Lancet. (1984) 1:1311–5. doi: 10.1016/s0140-6736(84)91816-6 6145023

[7] Wroblewski LE Peek RM Jr. Wilson KT. Helicobacter pylori and gastric cancer: factors that modulate disease risk. Clin Microbiol Rev. (2010) 23:713–39. doi: 10.1128/CMR.00011-10 PMC2952980 20930071

[8] Gan L He J Zhang X Zhang YJ Yu GZ Chen Y. Expression profile and prognostic role of sex hormone receptors in gastric cancer. BMC Cancer. (2012) 12:566. doi: 10.1186/1471-2407-12-566 23199240 PMC3517759

[9] Marshall B. Helicobacter pylori–a Nobel pursuit? Can J Gastroenterol. (2008) 22:895–6. doi: 10.1155/2008/459810 PMC2661189 19018331

[10] Khatoon J Rai RP Prasad KN. Role of Helicobacter pylori in gastric cancer: Updates. World J Gastrointest Oncol. (2016) 8:147–58. doi: 10.4251/wjgo.v8.i2.147 PMC4753165 26909129

[11] Chen CH Chung CY Wang LH Lin C Lin HL Lin HC. Risk of cancer among HIV-infected patients from a population-based nested case-control study: implications for cancer prevention. BMC Cancer. (2015) 15:133. doi: 10.1186/s12885-015-1099-y 25885746 PMC4369071

[12] Nie S Yuan Y. The role of gastric mucosal immunity in gastric diseases. J Immunol Res. (2020) 2020:7927054. doi: 10.1155/2020/7927054 32775468 PMC7396052

[13] Fridman WH. From cancer immune surveillance to cancer immunoediting: birth of modern immuno-oncology. J Immunol. (2018) 201:825–26. doi: 10.4049/jimmunol.1800827 30038034

[14] Ma ES Wang ZX Zhu MQ Zhao J. Immune evasion mechanisms and therapeutic strategies in gastric cancer. World J Gastrointest Oncol. (2022) 14:216–29. doi: 10.4251/wjgo.v14.i1.216 PMC8790417 35116112

[15] Lin H Lee E Hestir K Leo C Huang M Bosch E. Discovery of a cytokine and its receptor by functional screening of the extracellular proteome. Science. (2008) 320:807–11. doi: 10.1126/science.1154370 18467591

[16] Wang Y Colonna M. Interkeukin-34, a cytokine crucial for the differentiation and maintenance of tissue resident macrophages and Langerhans cells. Eur J Immunol. (2014) 44:1575–81. doi: 10.1002/eji.201344365 PMC4137395 24737461

[17] Greter M Lelios I Pelczar P Hoeffel G Price J Leboeuf M. Stroma-derived interleukin-34 controls the development and maintenance of langerhans cells and the maintenance of microglia. Immunity. (2012) 37:1050–60. doi: 10.1016/j.immuni.2012.11.001 PMC4291117 23177320

[18] Pandit J Bohm A Jancarik J Halenbeck R Koths K Kim SH. Three-dimensional structure of dimeric human recombinant macrophage colony-stimulating factor. Science. (1992) 258:1358–62. doi: 10.1126/science.1455231 1455231

[19] Chow A Brown BD Merad M. Studying the mononuclear phagocyte system in the molecular age. Nat Rev Immunol. (2011) 11:788–98. doi: 10.1038/nri3087 22025056

[20] Dai XM Ryan GR Hapel AJ Dominguez MG Russell RG Kapp S. Targeted disruption of the mouse colony-stimulating factor 1 receptor gene results in osteopetrosis, mononuclear phagocyte deficiency, increased primitive progenitor cell frequencies, and reproductive defects. Blood. (2002) 99:111–20. doi: 10.1182/blood.v99.1.111 11756160

[21] Li X Lei Y Gao Z Zhang B Xia L Lu J. Effect of IL-34 on T helper 17 cell proliferation and IL-17 secretion by peripheral blood mononuclear cells from rheumatoid arthritis patients. Sci Rep. (2020) 10:22239. doi: 10.1038/s41598-020-79312-z 33335239 PMC7746722

[22] Zwicker S Martinez GL Bosma M Gerling M Clark R Majster M. Interleukin 34: a new modulator of human and experimental inflammatory bowel disease. Clin Sci (Lond). (2015) 129:281–90. doi: 10.1042/CS20150176 PMC4557398 25896238

[23] Nian Z Dou Y Shen Y Liu J Du X Jiang Y. Interleukin-34-orchestrated tumor-associated macrophage reprogramming is required for tumor immune escape driven by p53 inactivation. Immunity. (2024) 57:2344–61 e7. doi: 10.1016/j.immuni.2024.08.015 39321806

[24] Mantovani A Allavena P Marchesi F Garlanda C. Macrophages as tools and targets in cancer therapy. Nat Rev Drug Discov. (2022) 21:799–820. doi: 10.1038/s41573-022-00520-5 35974096 PMC9380983

[25] Collison LW Workman CJ Kuo TT Boyd K Wang Y Vignali KM. The inhibitory cytokine IL-35 contributes to regulatory T-cell function. Nature. (2007) 450:566–9. doi: 10.1038/nature06306 18033300

[26] Vignali DA Collison LW Workman CJ. How regulatory T cells work. Nat Rev Immunol. (2008) 8:523–32. doi: 10.1038/nri2343 PMC2665249 18566595

[27] Collison LW Chaturvedi V Henderson AL Giacomin PR Guy C Bankoti J. IL-35-mediated induction of a potent regulatory T cell population. Nat Immunol. (2010) 11:1093–101. doi: 10.1038/ni.1952 PMC3008395 20953201

[28] Zhang J Lin Y Li C Zhang X Cheng L Dai L. IL-35 decelerates the inflammatory process by regulating inflammatory cytokine secretion and M1/M2 macrophage ratio in psoriasis. J Immunol. (2016) 197:2131–44. doi: 10.4049/jimmunol.1600446 27527600

[29] Xiong H Tang Z Xu Y Shi Z Guo Z Liu X. CD19(+)CD24(high)CD27(+) B cell and interleukin 35 as potential biomarkers of disease activity in systemic lupus erythematosus patients. Adv Rheumatol. (2022) 62:48. doi: 10.1186/s42358-022-00279-8 36494762

[30] Nakano S Morimoto S Suzuki S Tsushima H Yamanaka K Sekigawa I. Immunoregulatory role of IL-35 in T cells of patients with rheumatoid arthritis. Rheumatol (Oxford). (2015) 54:1498–506. doi: 10.1093/rheumatology/keu528 25731770

[31] Feng J Wu Y. Interleukin-35 ameliorates cardiovascular disease by suppressing inflammatory responses and regulating immune homeostasis. Int Immunopharmacol. (2022) 110:108938. doi: 10.1016/j.intimp.2022.108938 35759811

[32] Shao X Ma J Jia S Yang L Wang W Jin Z. Interleukin-35 suppresses antiviral immune response in chronic hepatitis B virus infection. Front Cell Infect Microbiol. (2017) 7:472. doi: 10.3389/fcimb.2017.00472 29181338 PMC5693856

[33] Hao S Chen X Wang F Shao Q Liu J Zhao H. Breast cancer cell-derived IL-35 promotes tumor progression via induction of IL-35-producing induced regulatory T cells. Carcinogenesis. (2018) 39:1488–96. doi: 10.1093/carcin/bgy136 30321288

[34] Sawant DV Yano H Chikina M Zhang Q Liao M Liu C. Adaptive plasticity of IL-10(+) and IL-35(+) T(reg) cells cooperatively promotes tumor T cell exhaustion. Nat Immunol. (2019) 20:724–35. doi: 10.1038/s41590-019-0346-9 PMC6531353 30936494

[35] Evans R Alexander P. Cooperation of immune lymphoid cells with macrophages in tumour immunity. Nature. (1970) 228:620–2. doi: 10.1038/228620a0 5529055

[36] Pittet MJ Michielin O Migliorini D. Clinical relevance of tumour-associated macrophages. Nat Rev Clin Oncol. (2022) 19:402–21. doi: 10.1038/s41571-022-00620-6 35354979

[37] Locati M Curtale G Mantovani A. Diversity, mechanisms, and significance of macrophage plasticity. Annu Rev Pathol. (2020) 15:123–47. doi: 10.1146/annurev-pathmechdis-012418-012718 PMC7176483 31530089

[38] Gordon SR Maute RL Dulken BW Hutter G George BM McCracken MN. PD-1 expression by tumour-associated macrophages inhibits phagocytosis and tumour immunity. Nature. (2017) 545:495–99. doi: 10.1038/nature22396 PMC5931375 28514441

[39] Mulder K Patel AA Kong WT Piot C Halitzki E Dunsmore G. Cross-tissue single-cell landscape of human monocytes and macrophages in health and disease. Immunity. (2021) 54:1883–900.e5. doi: 10.1016/j.immuni.2021.07.007 34331874

[40] Andon FT Digifico E Maeda A Erreni M Mantovani A Alonso MJ. Targeting tumor associated macrophages: The new challenge for nanomedicine. Semin Immunol. (2017) 34:103–13. doi: 10.1016/j.smim.2017.09.004 28941641

[41] Wei C Yang C Wang S Shi D Zhang C Lin X. Crosstalk between cancer cells and tumor associated macrophages is required for mesenchymal circulating tumor cell-mediated colorectal cancer metastasis. Mol Cancer. (2019) 18:64. doi: 10.1186/s12943-019-0976-4 30927925 PMC6441214

[42] Liu Q Zhang Y Zhang J Tao K Hambly BD Bao S. Inverse correlation between Interleukin-34 and gastric cancer, a potential biomarker for prognosis. Cell Biosci. (2020) 10:94. doi: 10.1186/s13578-020-00454-8 32765828 PMC7399616

[43] Cataldo JK Paul S Cooper B Skerman H Alexander K Aouizerat B. Differences in the symptom experience of older versus younger oncology outpatients: a cross-sectional study. BMC Cancer. (2013) 13:6. doi: 10.1186/1471-2407-13-6 23281602 PMC3576303

[44] Franze E Laudisi F Di Grazia A Maronek M Bellato V Sica G. Macrophages produce and functionally respond to interleukin-34 in colon cancer. Cell Death Discov. (2020) 6:117. doi: 10.1038/s41420-020-00350-7 33298879 PMC7644720

[45] Wang Q Zhang G An C Hambly BD Bao S. The role of IL-37 in gastrointestinal diseases. Front Immunol. (2024) 15:1431495. doi: 10.3389/fimmu.2024.1431495 39206201 PMC11349528

[46] Wang Q Ma L An C Wise SG Bao S. The role of IL-38 in intestinal diseases - its potential as a therapeutic target. Front Immunol. (2022) 13:1051787. doi: 10.3389/fimmu.2022.1051787 36405715 PMC9670310

[47] Munoz-Garcia J Cochonneau D Teletchea S Moranton E Lanoe D Brion R. The twin cytokines interleukin-34 and CSF-1: masterful conductors of macrophage homeostasis. Theranostics. (2021) 11:1568–93. doi: 10.7150/thno.50683 PMC7778581 33408768

[48] Zhang J Hu C Zhang R Xu J Zhang Y Yuan L. The role of macrophages in gastric cancer. Front Immunol. (2023) 14:1282176. doi: 10.3389/fimmu.2023.1282176 38143746 PMC10746385

[49] Sunderkotter C Goebeler M Schulze-Osthoff K Bhardwaj R Sorg C. Macrophage-derived angiogenesis factors. Pharmacol Ther. (1991) 51:195–216. doi: 10.1016/0163-7258(91)90077-y 1784630

[50] Klichinsky M Ruella M Shestova O Lu XM Best A Zeeman M. Human chimeric antigen receptor macrophages for cancer immunotherapy. Nat Biotechnol. (2020) 38:947–53. doi: 10.1038/s41587-020-0462-y PMC7883632 32361713

[51] Wang K Liu J Li J. IL-35-producing B cells in gastric cancer patients. Med (Baltimore). (2018) 97:e0710. doi: 10.1097/MD.0000000000010710 PMC5959432 29742730

[52] Bravo D Hoare A Soto C Valenzuela MA Quest AF. Helicobacter pylori in human health and disease: Mechanisms for local gastric and systemic effects. World J Gastroenterol. (2018) 24:3071–89. doi: 10.3748/wjg.v24.i28.3071 PMC6064966 30065554

[53] Wang Z Liu JQ Liu Z Shen R Zhang G Xu J. Tumor-derived IL-35 promotes tumor growth by enhancing myeloid cell accumulation and angiogenesis. J Immunol. (2013) 190:2415–23. doi: 10.4049/jimmunol.1202535 PMC3578001 23345334

[54] Li X Niu N Sun J Mou Y He X Mei L. IL35 predicts prognosis in gastric cancer and is associated with angiogenesis by altering TIMP1, PAI1 and IGFBP1. FEBS Open Bio. (2020) 10:2687–701. doi: 10.1002/2211-5463.13005 PMC7714063 33064893

[55] Yuan L Tan Z Huang J Chen F Hambly BD Bao S. Exploring the clinical significance of IL-38 correlation with PD-1, CTLA-4, and FOXP3 in colorectal cancer draining lymph nodes. Front Immunol. (2024) 15:1384548. doi: 10.3389/fimmu.2024.1384548 38533512 PMC10963446

[56] Chen F Zhang F Tan Z Hambly BD Bao S Tao K. Interleukin-38 in colorectal cancer: a potential role in precision medicine. Cancer Immunol Immunother. (2020) 69:69–79. doi: 10.1007/s00262-019-02440-7 31786620 PMC11027872

[57] Fan YG Zhai JM Wang W Feng B Yao GL An YH. IL-35 over-expression is associated with genesis of gastric cancer. Asian Pac J Cancer Prev. (2015) 16:2845–9. doi: 10.7314/apjcp.2015.16.7.2845 25854372

[58] Gu JH Wang XG Wang LQ Zhou LN Tang M Li P. Serum level of interleukin-35 as a potential prognostic factor for gastric cancer. Asia Pac J Clin Oncol. (2021) 17:52–9. doi: 10.1111/ajco.13403 33044052

